# BURDEN AMONG INFORMAL CAREGIVERS OF OLDER ADULTS IN GHANA: A SCOPING REVIEW

**DOI:** 10.1093/geroni/igae098.2479

**Published:** 2024-12-31

**Authors:** Joana Okine, Nicole Ruggiano

**Affiliations:** University of Alabama, Tuscaloosa, Alabama, United States; University of Alabama, Tuscaloosa, Alabama, United States

## Abstract

The aging population in Ghana has experienced a significant increase, expanding tenfold from 213,477 in 1960 to 1,991,736 in 2021 during the last population census. Projections indicate that by 2030, the population of persons aged 60 and above will hit 2.8 million (Mba, 2010). In Ghana, as in many African countries, kinship-based system of care is predominant as caregiving is viewed as a filial responsibility and an expression of love. There are empirical studies done in advanced countries like the USA that have documented the burden associated with caregiving for older adults. However, literature on burden among informal caregivers of older adults in Ghana in sparse. This study aimed to review available studies exploring caregiver burden experienced by informal caregivers in Ghana. We searched for studies published since 2013 across various databases, including Ebscohost (APA PsychInfo, CINAHL, Abstract in Social Gerontology, SocIndex, APA PsychArticles, Eric Ebscohost), Web of Science, Social Services Abstracts, Sociological Abstracts, Taylor and Francis, PubMed, and Google Scholar. Out of 505 identified studies, 9 met the inclusion criteria for this review. Findings suggest that caregivers of older adults in Ghana, irrespective of the care-recipient’s condition, face various burdens, including economic, physical, and psychological challenges. However, there is a noticeable gap in research addressing the burdens faced by informal Ghanian caregivers. It is crucial to focus on understanding and mitigating the burden on this group of caregivers to inform the development of culturally relevant interventions tailored to the needs of older adults and their caregivers.

